# Real-Time Machine Learning-Based Driver Drowsiness Detection Using Visual Features

**DOI:** 10.3390/jimaging9050091

**Published:** 2023-04-29

**Authors:** Yaman Albadawi, Aneesa AlRedhaei, Maen Takruri

**Affiliations:** 1 Department of Computer Science and Engineering, American University of Sharjah, Sharjah P.O. Box 26666, United Arab Emirates; 2 College of Engineering and Information Technology, Ajman University, Ajman P.O. Box 346, United Arab Emirates; 202211654@ajmanuni.ac.ae; 3 Center for Information, Communication and Networking Education and Innovation (ICONET), American University of Ras Al Khaimah, Ras Al Khaimah 72603, United Arab Emirates; maen.takruri@aurak.ac.ae

**Keywords:** driver drowsiness detection, eye aspect ratio, mouth aspect ratio, head pose estimation

## Abstract

Drowsiness-related car accidents continue to have a significant effect on road safety. Many of these accidents can be eliminated by alerting the drivers once they start feeling drowsy. This work presents a non-invasive system for real-time driver drowsiness detection using visual features. These features are extracted from videos obtained from a camera installed on the dashboard. The proposed system uses facial landmarks and face mesh detectors to locate the regions of interest where mouth aspect ratio, eye aspect ratio, and head pose features are extracted and fed to three different classifiers: random forest, sequential neural network, and linear support vector machine classifiers. Evaluations of the proposed system over the National Tsing Hua University driver drowsiness detection dataset showed that it can successfully detect and alarm drowsy drivers with an accuracy up to 99%.

## 1. Introduction

Drowsiness is a major concern with respect to road safety. Drivers’ unconsciousness due to microsleep can frequently lead to destructive accidents. Falling asleep at the wheel is usually related to lack of sleep, exhaustion, or mental health problems. In the UAE, the ministry of interior recorded 2931 car crashes in 2020. The number increased in 2021 to 3488 records. The majority of these traffic accidents were caused by distracted driving due to drowsiness, sudden swerving, or failure to maintain a safe distance between vehicles [1]. In this situation, it is crucial to exploit new technologies to plan and design systems that can track drivers and estimate their level of attention while driving. As multiple countries are concerned regarding this issue, researchers worldwide worked on building Driver Drowsiness Detection (DDD) systems that are capable of detecting drivers’ drowsiness signs in the early stages.

According to the literature, drowsiness detection systems can be grouped into three categories based on the measures that are used to detect the drowsiness signs [2,3,4,5]: biological-based, vehicle-based, and image-based systems. In the first category, biological-based measures rely on monitoring the body’s physiological signals including, ElectroEncephaloGraphy (EEG), ElectroCardioGraphy (ECG), ElectroMyoGraphy (EMG), Electro-OculoGraphy (EOG) signals, and blood pressure [6,7,8,9]. In this type of system, drowsiness is determined by detecting the signal’s deviation from the standard state’s characteristics and analyzing if the new signal indicates drowsiness. In the second category, vehicle-based measures depend on monitoring variations in the car’s movement patterns through different sensors’ installed to measure various vehicle and street parameters. To infer the drowsiness level, vehicle-based systems analyze the changes or abnormal behavior of the car, including, for example, the steering wheel angle, speed, or deviation from the lane [10,11]. The third category is the image-based measures which depend mainly on the drowsiness signs that appear on the driver’s face and head. These systems detect drowsiness by monitoring the drivers’ head movements and facial parameters such as the eyes, mouth facial expressions, eyebrows, or respiration [12,13,14].

All three categories have some limitations [2,15]. Biological-based systems can detect drowsiness in the initial stages due to their ability to compare the continuous changes in the physiological signals, but, in most biological-based systems, it is demanded that electrodes be connected to the driver’s body. This setup is usually inconvenient and uncomfortable for the driver. It also involves noise that affects the signal quality, leading to decreased accuracy. Vehicle-based systems depend generally on vehicle types, and can greatly be affected by multiple factors, including road characteristics, climate conditions, and the driver’s experience, habits, and ability to drive. Limitations of the image-based systems are strictly related to the quality of the camera used and its adaptability to different lighting conditions. The existence of objects covering parts of the face, such as glasses, sunglasses, masks, etc., can also affect the accuracy of image-based DDD systems. However, among these three systems, image-based systems are considered to be fully non-invasive, low cost, and minimally affected by road conditions. Therefore, image-based measures are widely deployed to develop versatile, affordable, real-time and, fully portable DDD devices [2,12,13,14,16,17].

In this work, we present a new image-based DDD system. It uses a unique combination of features derived from the driver’s facial parameters to train and test three classifiers, namely Random Forest (RF), sequential Neural Networks (NN), and linear Support Vector Machine (SVM). The features used in this system are Eye Aspect Ratio (EAR), Mouth Aspect Ratio (MAR), and head pose estimation. The proposed system is convenient for the driver in the sense that it does not require any sensors or equipment to be attached to the driver’s body. It is adaptable to be used in different vehicles, including buses, cars, motorcycles, and others. Evaluations of the proposed system on the National Tsing Hua University DDD (NTHUDDD) video dataset show that it can achieve accuracy up to 99%, indicating that it is an effective solution.

The rest of the paper is organized as follows: Section 2 summarizes the recent studies relating to the features used in this work. The methodology is presented in Section 3. Section 4 presents and discusses the results. The last section states the conclusions and the future directions.

## 2. Related Work

The problem of driver drowsiness detection has been studied by many researchers worldwide. The proposed approaches to tackle the problem can be mainly differentiated based on the drowsiness indicative features used [2]. Driver drowsiness indicative features obtained from body signs measurements (such as EEG, ECG, PPG, and EMG) are referred to as biological features, which, although accurate in detecting drowsiness, are inconvenient for the driver as they involve the use of sensors attached to the driver’s body [2,6,7,8,9]. Other widely used driver drowsiness indicative features are based on vehicle driving patterns where measurements such as the steering wheel angle and lane departure frequency are related to the driver drowsiness levels [2]. Although convenient for the driver, the literature shows that the accuracy of this method is not high [10,11]. The third drowsiness indicative features are image based. They are usually obtained from videos monitoring the driver’s behavior to extract features relating to the driver’s eye, mouth, and head movements [2]. They are more convenient for the driver than the biological-based ones as they do not involve attaching equipment or sensors to the driver’s body.

Image-based systems are the most commonly used techniques for detecting driver drowsiness. Facial parameters such as the eyes, mouth, and head can be used to identify many visual behaviors that fatigued people exhibit. Such drowsy behaviors can be recorded by cameras or visual sensors. Then, from these records, several features can be extracted, and by using computer vision techniques they are analyzed to visually observe the driver’s physical condition in order to detect drowsiness in a non-invasive manner. Broadly, image-based systems are categorized into three categories depending on the observation of the eyes, mouth, and head movements [2]. Various image-based features have been used in the literature. These include blink frequency, maximum duration of closure of the eyes [13], percentage of eyelid closure [18], eye aspect ratio [19], eyelids’ curvature [17], yawning frequency [20], MAR [21], mouth opening time [22], head pose [23], head-nodding frequency [4], and head movement analysis [24]. Combinations of these features have been considered as well [20,21,25]. In this section, we provide a detailed explanation of the features that are used in our proposed system.

The most common features used to detect drowsiness in image-based systems are extracted from the eye region. Several researchers proposed the EAR [26,27,28] as a simple metric to detect eye blinking using facial landmarks. It is utilized to estimate the eye openness degree. A sharp drop in the EAR value leads to a blink being recorded.

Maior et al. [27] developed a drowsiness detection system based on the EAR metric. They calculated the EAR values for consecutive frames and used them as inputs for machine learning algorithms including the multilayer perceptron, RF, and SVM classification models. Their evaluation results showed that the SVM performed the best with 94.9% accuracy. The EAR metric was also used in [29], who explored drowsiness as an input for a binary SVM classifier. The model detected the driver’s drowsiness state with 97.5% accuracy.

Mouth behavior is a good indicator of drowsiness as it provides useful features for DDD. In [30], the authors proposed to track mouth movement to recognize yawning as a drowsiness indicator. In their experiment, they used a dataset of 20 yawning images and over 1000 normal images. The system used a cascade classifier to locate the driver’s mouth from the face images, followed by an SVM classifier to identify yawning and alert the driver. The final results gave a yawning detection rate of 81%. Another mouth-based feature is the mouth opening ratio [29]. It is also referred to as the MAR [21]. It describes the opening degree of the mouth as an indicator for yawning. This feature was fed to an SVM classifier in [29], achieving an accuracy of 97.5%.

Another useful parameter for detecting drowsiness in image-based systems is head movements which can signal drowsy behavior. Accordingly, they can be used to derive features that are useful for detecting drowsiness using machine learning. Such head features include head-nodding direction, head-nodding frequency [4], and head pose [31]. In [31], the forehead was used as a reference to detect the driver’s head pose. Infrared sensors were used in [24] to follow the head movement and detect the driver’s fatigue. In [32,33], before head position analysis was performed, a special micro-nod detection sensor was used in real-time to track the head pose feature in 3D.

Moujahid et al. [20] presented a face-monitoring drowsiness-detection system that captured the most prominent drowsiness features using a hand-crafted compact face texture descriptor. Initially, they recorded three drowsiness features, namely head nodding, yawning frequency, and blinking rate. After that, they applied pyramid multi-level face representation and feature selection to achieve compactness. Lastly, they employed a non-linear SVM classifier that resulted in an accuracy of 79.84%.

Dua et al. [34], introduced a driver drowsiness-detection architecture that used four deep learning models: ResNet, AlexNet, FlowImageNet, and VGG-FaceNet. These models are extracted from the driver’s footage features that include head gestures, hand gestures, behavioral features (i.e., head, mouth, and eye movements), and facial expressions. Simulated driving videos were fed to the four deep learning models. The outputs of the four models were fed to a simple averaging ensemble algorithm followed by a SoftMax classifier, which resulted in 85% overall accuracy.

## 3. Methodology

The methodology followed to develop the proposed DDD system is presented in detail in this section. Firstly, the system design is illustrated. Secondly, a dataset description is provided. Lastly, the four main steps followed in the implementation process are discussed, which are (1) preprocessing, (2) feature extraction, (3) data labeling, and (4) classification.

### 3.1. System Design

**Figure 1:**
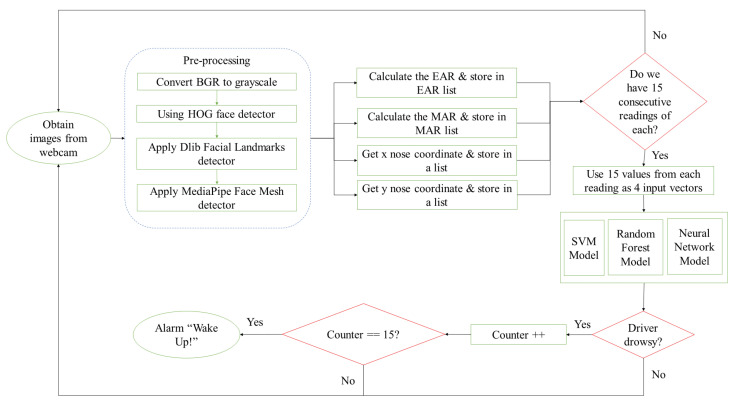
System design.

The flowchart in Figure 1 shows the design flow of the proposed drowsiness-detection system. The system design consists of five main steps. In the first step, the system starts by capturing a video that monitors the driver’s head and extracts frames from it. The second step is preprocessing, where first, the Blue, Green, and Red (BGR) colored frames are each converted to grayscale. Then, for the eyes and mouth region, face detection is applied by utilizing the Dlib Histogram of Oriented Gradients (HOG) face detector [35]. The Dlib facial landmarks detector is then applied to extract the eyes and mouth regions. Lastly, in the preprocessing step, to capture the head region, MediaPipe face mesh [36] is used to obtain a 3D map of the face and extract the 3D nose coordinates to use as a reference to estimate the driver’s head position.

The third step involves calculating for each frame a feature vector containing the EAR, MAR, and the nose X–Y coordinates, and storing them in a separate list. This is repeated to populate a window (matrix) with feature vectors corresponding to 15 consecutive frames. Once the system has the first 15 feature vectors stored, it feeds them to the trained classification model which results in initial drowsy or alert labels. The final decision of whether the driver is drowsy is taken if the drowsy label is produced 15 consecutive times and an alarm will sound to alert the driver. Otherwise, the driver will be considered alert. As the process continues, the system employs the moving window concept. The moving window is fixed in size and can only take 15 feature vectors corresponding to a matrix of dimension 4 × 15. When a new frame is recorded, its corresponding feature vector is fed into the feature window while the oldest feature vector in the window is dropped out.

Accordingly, the first decision about the driver drowsiness status is given by the system after 1 s, as the system waits to populate the window with 15 feature vectors, followed by counting 15 classifiers labels; i.e., the first decision requires recording 30 frames: 15 to populate the feature window, and 15 label counts. Referring to the moving window discussed above, the following decisions, in contrast, are taken almost instantly. When a new frame is recorded, its corresponding feature vector is fed into the feature window while the oldest feature vector in the window is dropped out. In this case, we have now a full window with 15 feature vectors and 14 previous labels, and the current (new) label which accounts for a time period of 1 frame (1/30 s = 33 ms). A new decision requires the introduction of one new frame which spans 33 ms. Therefore, considering that the preprocessing time and the classification times are minimal, our system’s first decision takes 1 s, while the following decisions will be reported every 33 ms, indicating that the response can be considered as being in real time.

### 3.2. Dataset

In this work, the NTHUDDD video dataset was used to implement this DDD system [37]. The dataset was obtained under simulated driving conditions. A total of 36 subjects were recorded while sitting on a chair playing a driving game with a simulated driving wheel and pedals, with their facial expressions monitored for drowsiness signs. Active infrared (IR) illumination was used to acquire IR videos in the dataset collection. The videos under consideration in this work were taken at a rate of 30 frames/s with a resolution of 640 × 480 pixels and an overall length of 9 h and a half. They were recorded in AVI format.

**Figure 2:**
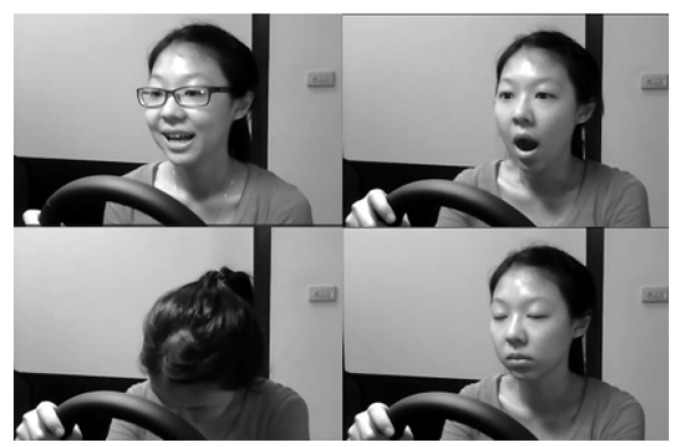
Drivers’ behaviors.

The 36 subjects were of various ethnicities, genders, and facial characteristics. They were recorded under different scenarios with and without glasses or sunglasses under a variety of simulated driving conditions during the day and night times. Various subject behaviors were recorded including normal driving, talking, turning around, slow eye blinking, yawning, and head nodding. Figure 2 shows some of these behaviors. Table 1 illustrates a further description of the dataset. This work has utilized 23 subjects from the NTHUDDD dataset: 18 for training and 5 for testing. The subject selection was based on the different facial appearances and scenarios including wearing/not wearing eyeglasses.

### 3.3. Preprocessing

**Figure 3:**
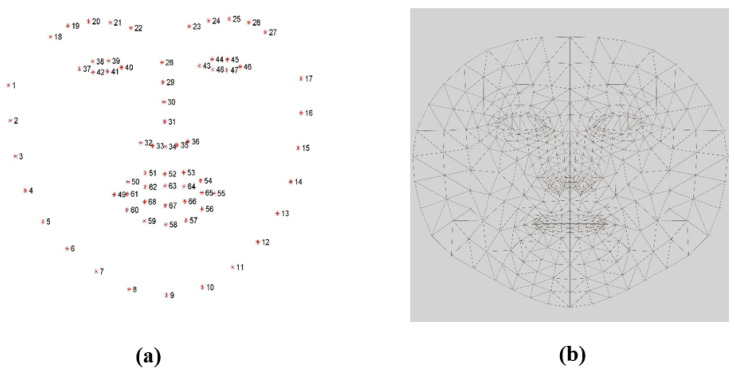
The map of the two landmarks solutions that were used. (**a**) Dlib facial landmarks solution map. (**b**) MediaPipe face mesh solution map.

For preprocessing, the colored frames are each converted to grayscale. Then, to obtain the eyes and mouth features, the face was extracted by utilizing Dlib’s HOG face detector, where the detector function returned a rectangle’s coordinates, which surround the face region. Following that, the Dlib facial landmarks solution was utilized. This solution estimates the location of 68 points on the face, forming a map that represents the key facial structures on the face, as shown in Figure 3a [19]. Thus, it was used to detect and extract the eye and mouth regions.

For the head pose estimation feature, we used the MediaPipe face mesh solution [36], which is a face geometry solution that is used to estimate 468 face landmarks in 3 dimensions, as shown in Figure 3b. The X and Y output coordinates of the face mesh solution are normalized based on the frame size. While the z coordinate represents the face mesh depth which reflects the distance of the head from the camera. In order to estimate the head pose in the captured video, the initial nose coordinates were first extracted to be used as a reference for the head location and movements in the following frames.

### 3.4. Feature Extraction

Various human and vehicle features were used to model different drowsiness detection systems. However, in this work, the modeling is based on the EAR and MAR metrics along with drowsy head pose estimation.

#### 3.4.1. EAR Metric

**Figure 4:**
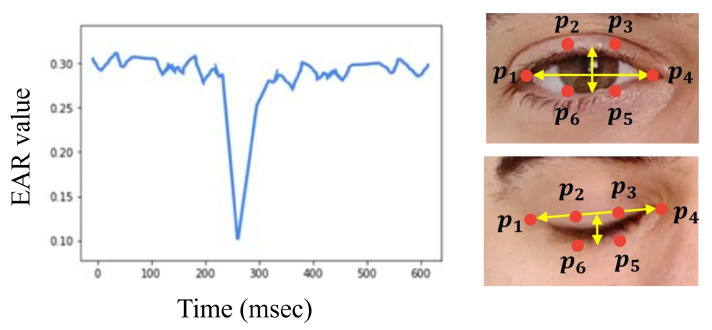
EAR change over time [38].

According to Rosebrock [19], detecting blinking using the EAR feature has multiple advantages compared to detection with traditional image-processing methods. In traditional methods, first eye localization is applied. Then, thresholding is used to find the whites of the eyes in the image. Following that, eye blinking is indicated by detecting the disappearance of the eye’s white region. In contrast, no image processing is needed when using the EAR metric. Thus, using it will require less memory space and processing time. Instead, the EAR feature depends on calculating the ratio of the distance between eyes’ facial landmarks, which makes it a straightforward solution. In general, the EAR metric computes a ratio extracted from the horizontal and vertical distances of six eye landmark coordinates, as shown in Figure 4 [38]. These coordinates are numbered from the left eye corner starting from p1 and revolving clockwise to p6. Rosebrock [19] explains that all six coordinates from p1 to p6 are two-dimensional. According to [39], in the case of open eyes, the EAR value remains approximately constant. However, if the eyes were closed, the difference between coordinates p3 and p5 and p2 and p6 demolishes; thus, the EAR value drops down to zero, as illustrated in Figure 4.

In order to extract the EAR feature, Equation (1) was utilized. As shown in the equation below, to compute the EAR ratio value, the numerator calculates the distance between the vertical landmarks. While the denominator calculates the distance between the horizontal landmarks and multiplies it by two to balance it with the nominator [39]. By utilizing Equation (1), the EAR values were calculated for each frame and stored in a list.

(1) $$EAR=\frac{\left|\left|p2-p6\right|+\left|p3-p5\right|\right|}{2\left|p1-p4\right|}$$

#### 3.4.2. MAR Metric

**Figure 5:**
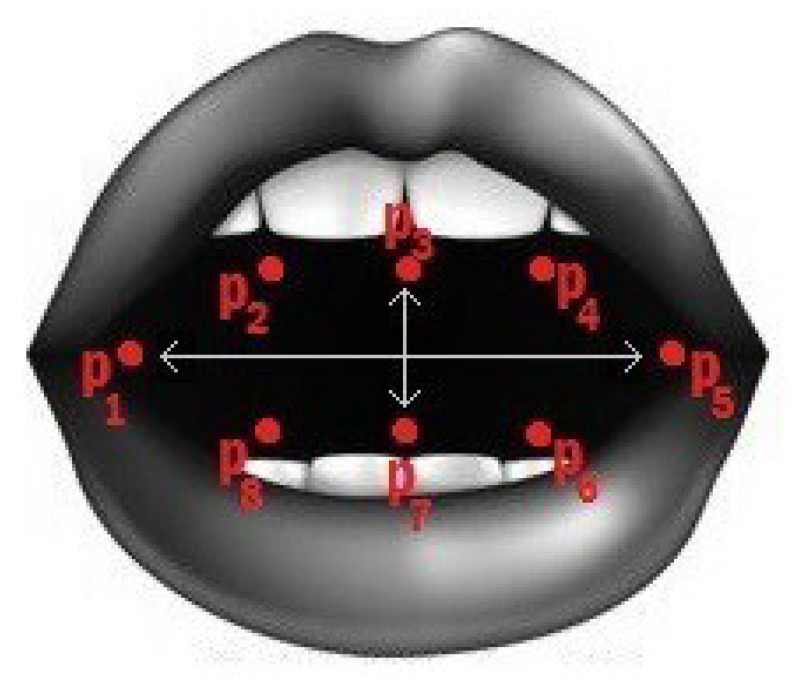
The 8 coordinates used to calculate the MAR [40].

**Figure 6:**
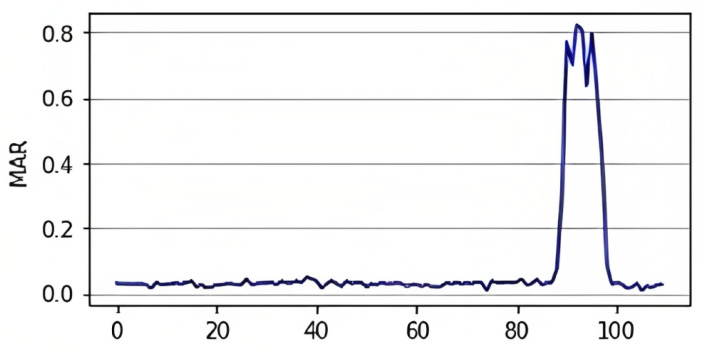
MAR change over time [40].

Similar to the EAR, the mouth aspect ratio, or MAR, is used to calculate the openness degree of the mouth. In this facial landmark, the mouth is characterized by 20 coordinates (from 49 to 68), as shown in Figure 3a. However, we used points from 61 to 68, as displayed in Figure 5, to obtain the mouth openness degree. Using these coordinates, the distance between the top lip and the bottom lip is calculated using (2) to determine whether the mouth is open or not [40]. In (2), the numerator calculates the distance between the vertical coordinates, and the denominator calculates the distance between the horizontal coordinates. Similarly to (1), the denominator is multiplied by two to balance it with the nominator. As shown in Figure 6, increasing the value of the MAR indicates the mouth is open.

(2) $$MAR=\frac{\left|p2-p8\right|+\left|p3-p7\right|+\left|p4-p6\right|}{2\left|p1-p5\right|}$$

#### 3.4.3. Drowsy Head Pose

In this work, head pose estimation was achieved by finding the rotation angle of the head. The rotation angle can be defined as the amount of rotation of an object around a fixed point referred to as the point of rotation. To find the rotation angle of the head, first, the center nose landmark was acquired using MediaPipe face mesh for use as a reference and as the point of rotation for the head position in the frame, as mentioned earlier in preprocessing. Then, the nose’s X and Y landmarks were normalized by multiplying them by the frame width and height, respectively. Following that, by taking the initial nose 3D coordinates as the point of rotation, the rotation angles of the X and Y axis are calculated and used to estimate if the head position is up, down, left, or right based on a set of thresholds. We have estimated the angle thresholds as follows:

- Head pose up, if X of angle 7${}^{{}^{\circ}}$
- Head pose down, if X of angle −7${}^{{}^{\circ}}$
- Head pose right, if Y of angle 7${}^{{}^{\circ}}$
- Head pose left, if Y of angle −7${}^{{}^{\circ}}$

### 3.5. Data Labeling

According to [39,41], blinking is a quick movement of closing and reopening the eyes, which approximately takes between 100 to 400 ms, while yawning is a quick act of opening and closing the mouth, which lasts for around 4 to 6 s. As for a drowsy head pose, it can be described as random head titling due to severe drowsiness that is usually associated with eye closure, and it may last for a few seconds. Blinking, yawning, and head pose patterns differ depending on the person, action duration, degree of opening or closure, degree of head tilting, and speed. Moreover, one reading of EAR, MAR, and X and Y nose coordinates per frame is not enough to capture the event of blinking, yawning, or drowsy head pose. Thus, in order to detect the different drowsy action patterns, we have used four fifteen-frame length vectors, for each of the four readings, consecutively, as an input to the classifiers.

It is well known that when a person starts feeling sleepy that the eye-closing time becomes longer. As a result, we label in this work a blink of 400 ms or longer as indicative of a drowsy driver. Given that the videos were taken at a frame rate of 30 frames/s, i.e., the frame time is 1/30 s, then a drowsy blink will span at least 13 frames. Taking into consideration that people can statistically vary in their eye closure time when they start feeling sleepy, we relax the 400 ms to 500 ms, which spans 15 frames, as was the case in [42].

In order to verify our assumption, we tested different temporal window sizes during the labeling phase, including 9, 13, 15, 17, and 21 frames (see Table 2). By doing that, we aimed to experimentally figure out the number of frames that better capture the different events of eye closure, yawning, and drowsy head pose. Our tests were conducted on three randomly labeled subjects from our training dataset. As shown in Table 2, smaller windows resulted in detecting more drowsy cases because short eye blinks (less than 400 ms) were considered as drowsy while they are, in fact, not drowsy. On the other hand, long windows resulted in some real drowsy cases being missed or not detected. The results reported in the table supported our initial decision of using a 15-frame-long temporal window as it is the case that mostly matched the video drowsiness labels. Consequently, a window of 15 frames in length was adopted.

This temporal window was used to prepare the input data as follows: for every 30 frames/s video, the MAR value of the Nth frame is calculated and stored in a list, along with the MAR values from the N − 7 and N + 7 frames. Following that, these 15 MAR values are concatenated, forming a 15-dimensional feature vector for that Nth frame. In this case, we are taking the 7 neighboring frames (from each side) for each Nth frame in order to capture the actual state of the mouth at that frame, either close or open. The same method was applied to prepare the EAR, x, and y nose coordinates input vectors, resulting in a final input of four 15-frame long input vectors.

Labeling the training input data was a two-step process, where first, the eyes, mouth, and head state are labeled separately. Then, a final label of the driver’s state was given. As for the eyes, an EAR threshold of 0.2 was set to reflect if the drivers’ eyes were open or not. For the mouth, the MAR was given a 0.5 threshold to indicate if the mouth was wide open. In terms of the head, nose coordinates were given a set of angle thresholds to reflect the different poses that a drowsy driver’s head may position at, as explained previously. After labeling the state of these three parts, a final label was given of either 0 (alert) or 1 (drowsy) to indicate if the driver was drowsy or not. Label 1 was given if either of these states were met, or if a closed eye, open mouth, or drowsy head pose was present.

When choosing the thresholds, we studied the maximum EAR (MAX EAR) and maximum MAR (MAX MAR) of different eyes and mouth shapes and sizes in the 18 subjects from our training dataset, as shown in Table 3. MAX EAR reflects the EAR value at the regular openness state of the eyes, and MAX MAR reflects the maximum MAR value that takes place when yawning. We found out that most of the subjects have a MAX EAR range between 0.3 and 0.37. However, we still need to consider the cases of subjects with small eyes, whose MAX EAR value reached a minimum of 0.23. Thus, we experimented with different thresholds during the labeling stage, as illustrated in Table 4. According to Table 4, at a threshold value greater than 0.4, all data frames of all the subjects were labeled “Closed eyes” regardless of the eye state, as none of the subjects in the training dataset has a MAX EAR greater than 0.37. Threshold values between 0.35 and 0.25 had a similar issue as they did not work with subjects of MAX EAR value of 0.34 and below. At the threshold value of 0.2, all the subjects got labels of “Open eyes” or “Closed eyes” successfully without any bias. Lastly, threshold values that were less than 0.2 worked as well, but they reduced the “Closed eyes” labels in the training dataset. Thus, taking into consideration both subjects with small eyes and having a balanced training dataset, we decided to set an EAR threshold value of 0.2 to identify the drowsy eyes from the alert.

Similarly, for the MAX MAR values, we noticed in Table 3 that the majority of the drivers reach a MAX MAR value of 0.9 when yawning. However, drivers with small mouths can reach a MAX value of 0.6 or 0.7 depending on the size of the mouth and the way of yawning. Thus, we applied some experiments during the labeling stage to choose the best MAR threshold, as shown in Table 5. According to Table 5, at a threshold value greater than 0.9, all data frames of all the subjects were labeled “Closed mouth” regardless of the mouth state, as the MAX MAR value for the subjects in the training set is 0.9. For threshold values between 0.8 and 0.6, we noticed a similar issue as the frames of subjects with MAX MAR of 0.79 or below were always labeled as “Closed mouth.” At the threshold value of 0.5, we have successfully labeled all subjects with a label “Open mouth” or “Closed mouth,” reflecting the true state of the mouth. Any threshold value below 0.5 caused some frames to be mislabeled in cases such as talking or laughing. Therefore, we decided to set the MAR threshold to a minimum value of 0.5 to address any unique cases.

### 3.6. Classification

After labeling the extracted values, two main machine learning data preprocessing steps were performed. First, data balancing is an essential step when dealing with unbalanced instances between the two classes. In our case, there were 300,266 non-drowsy labeled as 0 cases and 72,658 drowsy cases labeled as 1. Using under-sampling and over-sampling from the imbalanced learning library, we over-sampled the minority class (labels 1) and under-sampled the majority class (labels 0).

The second preprocessing step is data splitting, where a data splitting function from the scikit-learn library was utilized. The data were split into 70% training and 30% testing. The training data was used to train and create the models, while the testing data was utilized to test the performance of the models. After splitting the dataset, three classification models were applied: RF, sequential NN [43], and SVM. Then, the parameters of the three models were tuned and optimized by utilizing grid search hyperparameters [28].

Random forest (RF) is a popular and effective machine learning algorithm, created by Breiman [44]. It involves constructing a group of decision trees that work together to make predictions. The trees are created using bootstrap samples and randomly selecting variables at each node. The RF model combines the predictions of each tree to determine the final prediction. In this study, the scikit-learn library’s RF classifier was used with “entropy” as the criterion parameter and 50 trees in the forest.

The sequential neural network (NN) model, also known as the feedforward neural network, is the basic type of neural network model [43]. In this study, we used the Keras library to build our neural network model. Keras offers an easy way to build models using the sequential approach, where each layer is added one at a time with weights corresponding to the following layer. In this work, a neural network with six layers was created, consisting of an input layer, four hidden layers with five nodes each using ReLU activation, and an output layer with one node using sigmoid activation. The model classifies the output as either 1 for drowsy or 0 for nondrowsy.

Support vector machine (SVM) [45] is a supervised machine learning model that classifies two groups of data by finding a hyperplane in N dimensions. The goal is to select the hyperplane that maximizes the margin between data points, which improves future classification accuracy. The SVM model is popular because it has low computational complexity and high accuracy. The support vector classification (SVC) from the scikit-learn library was used with a linear kernel, a regularization parameter of C = 1, probability estimates enabled, and the random state parameter was set to 0 to control data shuffling.

## 4. Results and Discussion

This section lists the specifications of our development environment. In addition, it presents and discusses the results of the trained models using the testing data that was extracted from the NTHUDDD dataset. By finding the confusion matrix, accuracy, sensitivity, specificity, macro precision, and macro F1-score, and through two visual plots of the results, the best model for drowsiness detection was determined. This section also compares the results of the proposed system with other DDD systems.

While implementing this system, we used a laptop equipped with an i7 processor, 16 GB RAM, and an integrated GPU (Intel(R) UHD Graphics 620). As for the development environment, we used Jupyter Notebook in Anaconda and developed the system using Python 3.7. We mainly used scikit-learn 1.1, TensorFlow 2.12, Keras 2.12, Dlib 19.24.1, OpenCV 4.7.0, and MediaPipe 0.9.3.0 libraries and packages.

The implementation was performed in two steps, namely, the training step and the testing step. In the training step, the model was trained offline on the precollected NTHUDDD standard dataset. In the testing step, the video footage of the driver’s face was taken at 30 frames/s by a webcam fixed at the center of the car’s dashboard. The webcam fed the video frames to a laptop that was preloaded with the trained DDD model. The trained DDD model extracted the feature vector corresponding to each frame and classified it in a time period of (2–4 ms), which is negligible compared to the 33 ms time span between one frame and the other, thus, making the decision mainly dependent on the frame time (33 ms) and meaning it can therefore can be considered a real-time decision system.

Table 6 illustrates the results of the trained models. The results show that the best performance is achieved by the RF model. When analyzing the results, it is evident that the RF model gave an almost perfect performance as it achieved 99% in accuracy, sensitivity, specificity, macro precision, and macro F1-score. In terms of the performance of the sequential NN model, it achieved second-best results with 96% accuracy, 97% sensitivity, and 96% specificity, macro precision & macro F1-score. As for the SVM model, it achieved the lowest results, where it showed 80% accuracy, 70% sensitivity, and 88% specificity.

**Figure 7:**
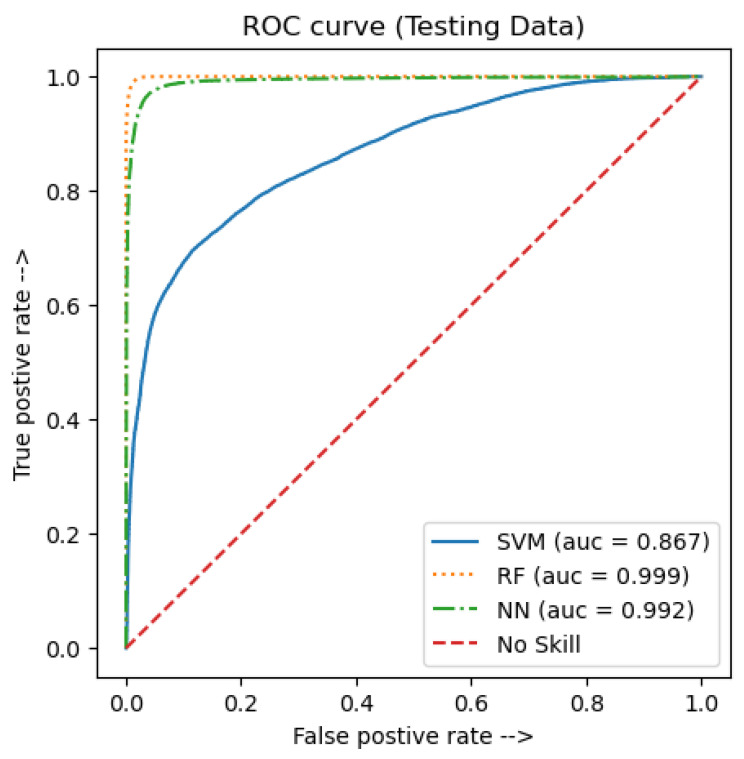
ROC curve for the testing data.

**Figure 8:**
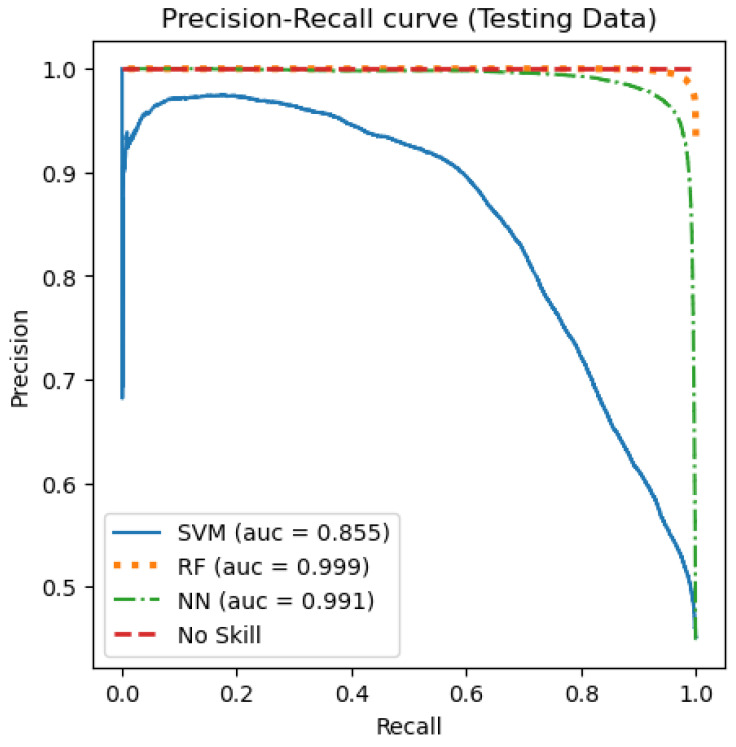
The precision–recall curve for the testing data.

Figure 7 and Figure 8 show the precision, recall, and the Receiver Operating Characteristic (ROC) curves. Those curves were plotted as means of visualizing the three models’ performance. As for the ROC curve, it is usually used for binary classification models to describe its performance by showing if the model predicts the outcome as a positive class when it is actually positive [46].

A score called Area Under the Curve (AUC) can be calculated to reflect the total area under the ROC curve and the separability degree. It is important to note that if a model shows a high AUC value, then it is better at predicting the actual outcomes of the true negative and true positive classes. The ROC curve for the testing data is presented in Figure 7. Similarly, the precision–recall curve is a perfect evaluating tool for binary classification models [46]. In this curve, if a model showed a high AUC score, that indicates a better predicting performance. Figure 8 shows the precision–recall curve for the testing data. As can be seen in Figure 7, both the RF and sequential NN models achieved a high AUC score. However, the AUC score of the SVM model was noticeably lower, which reached 0.867. Regardless, when comparing the three curves, it can be seen that the best performance was achieved by the RF model, with a 0.999 AUC score. Likewise, looking at Figure 8, the RF model gave an AUC score of 0.999, which reflects the best performance, compared to both the sequential NN and the SVM models, which gave a score of 0.991 and 0.855, respectively.

The above discussion clearly shows that our proposed system can differentiate drowsy drivers from alert ones. It is easy to use and convenient for the drivers as it is non-invasive, non-intrusive, and does not require any sensors or equipment to be attached to the driver’s body. It is also adaptable to be used in different vehicles, including buses, trucks, cars, motorcycles, and construction vehicles. Table 7 presents the most recent literature on drowsiness-detection systems. Due to the different utilization of the datasets and the features, one-to-one comparison is not applicable. However, as illustrated, our RF model outperforms the other techniques available in the literature. Nevertheless, it is important to note that the system has some limitations. The HOG face detector can fail in some scenarios. Some of these include having more than one subject in the frame, variation in the intensities while driving, and driving on a dark street.

## 5. Conclusions

In conclusion, in this paper, we proposed a real-time image-based drowsiness-detection system. In order to implement drowsiness detection, a webcam was used to detect the driver in real time and extract the drowsiness signs from the eyes, mouth, and head. Then three classifiers were applied at the final stage. When a drowsiness sign is detected, an alarm sounds, alerting the driver and ensuring road safety. Evaluation of system performance over the NTHUDDD dataset resulted in an accuracy of 99% for the RF classifier.

In the future, we plan to develop a mobile application to allow users to easily use the system while driving. Furthermore, to overcome the limitation of the HOG face detector, we intend to use a more advanced camera that can adapt to the changes in lighting intensity and automatically detect and focus on the driver’s face.

## Figures and Tables

**Table 1:** NTHUDDD dataset description.

Driver’s Behaviors	Description
Looking aside	When the head turns left or right
Talking and laughing	When talking or laughing
Sleepy-eyes	When eyes slowly close due to drowsiness
Yawning	When mouth open wide due to drowsiness
Nodding	When head falls forward when drowsy
Drowsy	When the driver visually looks sleepy, showing signs such as slowly blinking, yawning, and nodding
Stillness	When normally driving
**Videos**	**Description**
yawning.avi	Contains yawning behavior
sleepyCombination.avi	Contains a combination of drowsy behaviors (nodding, slow eye blinking, and yawning)
slowBlinkWithNodding.avi	Contains slow eye blinking and head nodding behavior
nonsleepyCombination.avi	Contains a combination of non-drowsy behaviors talking, laughing, looking aside

**Table 2:** Varying the temporal window size while labeling the training dataset.

Temporal Window Size	Training Instances Labeled Drowsy in Subject 1	Training Instances Labeled Drowsy in Subject 2	Training instances Labeled Drowsy in Subject 3
9	2964	2218	3924
13	2851	2111	3802
15	2800	2057	3768
17	2739	2010	3714
21	2645	1922	3655

**Table 3:** MAX EAR and MAR values for the training set subjects.

Subject Number	Max EAR	Max MAR
1	0.36	0.75
2	0.36	0.9
3	0.23	0.9
4	0.34	0.9
5	0.36	0.9
6	0.34	0.6
7	0.32	0.8
8	0.3	0.9
9	0.29	0.9
10	0.35	0.9
11	0.31	0.9
12	0.3	0.9
13	0.25	0.8
14	0.28	0.9
15	0.24	0.6
16	0.37	0.9
17	0.34	0.55
18	0.36	0.9

**Table 4:** EAR threshold experimental testing while labeling the training dataset.

Training Video	EAR Thresholds	Results after Labeling the Training Data
	>0.4	All data frames of all the subjects were labeled “Closed eyes”
	0.35	All data frames of subjects with MAX EAR of 0.34 or less were labeled “Closed eyes”
	0.3	All data frames of subjects with MAX EAR of 0.29 or less were labeled “Closed eyes”
**sleepy Combination.avi** **and** **slowBlinkWith** **Nodding.avi**	0.25	All data frames of subjects with MAX EAR of 0.24 or less were labeled “Closed eyes”
	0.2 *	**All data frames of all subjects were labeled** **as “Open eyes” or “Closed eyes” successfully**
	<0.2	Data frames of all subjects were labeled “Open eyes” in most cases

* Chosen EAR threshold value is in bold.

**Table 5:** MAR threshold experimental testing while labeling training dataset.

Training Video	MAR Thresholds	Results after Labeling the Training Data
	>0.9	All data frames of all the subjects were labeled “Closed mouth”
	0.8	All data frames of subjects with MAX MAR of 0.79 or less were labeled “Closed mouth”
	0.7	All data frames of subjects with MAX MAR of 0.69 or less were labeled “Closed mouth”
**yawning.avi** **and** **nonsleepy** **Combination.avi**	0.6	All data frames of subjects with MAX MAR of 0.59 or less were labeled “Closed mouth”
	0.5 *	**All data frames of all subjects** **were labeled as “Open mouth”** **or “Closed mouth” successfully**
	<0.5	Data frames of all subjects were labeled “Open mouth” in cases where the driver is talking/laughing

* Chosen MAR threshold value is in bold.

**Table 6:** Results of the proposed DDD system.

	Accuracy	Sensitivity	Specificity	Macro Precision	Macro F1-Score
**Linear SVM**	0.80	0.70	0.88	0.80	0.79
**RF**	0.99	0.99	0.98	0.99	0.99
**Sequential NN**	0.96	0.97	0.96	0.96	0.96

**Table 7:** Comparison of the proposed method with similar techniques.

Method	Year	Dataset	Feature	Algorithm	Accuracy
[47]	2018	Custom	Eye and Mouth	Logistic regression	92%
[48]	2019	Custom	Eye	CNN	96.42%
[25]	2019	NTHUDDD dataset	Eye and Mouth	Gamma fatigue detection network	97.06%
[49]	2019	NTHUDDD dataset	Eye, Mouth, and Head	3D convolutional networks	76.2%
[50]	2020	Custom	Eye	SVM and AdaBoost	SVM: 96.5%, AdaBoost: 95.4%
[51]	2020	NTHUDDD dataset	Eye, Mouth, and Head	3D convolutional networks	92.19%
[34]	2021	NTHUDDD dataset	Eye, Mouth, and Head	CNN	85%
[52]	2021	Custom	Eye and Body motion	SVM	90%
**Proposed System**	2022	NTHUDDD dataset	Eye, Mouth, and Head	RF, SVM, and Sequential NN	RF: 99%, SVM: 80%, Sequential NN: 96%

## Data Availability

Not applicable.

## Abbreviations

The following abbreviations are used in this manuscript:

DDD	Driver Drowsiness Detection
EEG	electroencephalography
ECG	electrocardiography
EMG	electromyography
EOG	electrooculography
RF	Random Forest
NN	Neural Networks
SVM	Support Vector Machine
BGR	Blue Green Red
EAR	Eye Aspect Ratio
MAR	Mouth Aspect Ratio
NTHUDDD	National Tsing HuaUniversity DDD
CNN	Convolutional Neural Networks
HOG	Histogram of Oriented Gradients
ROC	Receiver Operating Characteristic
AUC	Area Under the Curve

## References

[1] Al Amir S. Road Accidents in UAE Caused 381 Deaths Last Year. https://www.thenationalnews.com.

[2] Albadawi Y., Takruri M., Awad M. (2022). A review of recent developments in driver drowsiness detection systems. Sensors.

[3] Ramzan M., Khan H.U., Awan S.M., Ismail A., Ilyas M., Mahmood A. (2019). A survey on state-of-the-art drowsiness detection techniques. IEEE Access.

[4] Sikander G., Anwar S. (2018). Driver fatigue detection systems: A review. IEEE Trans. Intell. Transp. Syst..

[5] Pratama B.G., Ardiyanto I., Adji T.B. A review on driver drowsiness based on image, bio-signal, and driver behavior. Proceedings of the IEEE 2017 3rd International Conference on Science and Technology-Computer (ICST).

[6] Kaur R., Singh K. (2013). Drowsiness detection based on EEG signal analysis using EMD and trained neural network. Int. J. Sci. Res..

[7] Kundinger T., Sofra N., Riener A. (2020). Assessment of the potential of wrist-worn wearable sensors for driver drowsiness detection. Sensors.

[8] Sahayadhas A., Sundaraj K., Murugappan M., Palaniappan R. (2015). Physiological signal based detection of driver hypovigilance using higher order spectra. Expert Syst. Appl..

[9] Khushaba R.N., Kodagoda S., Lal S., Dissanayake G. (2010). Driver drowsiness classification using fuzzy wavelet-packet-based feature-extraction algorithm. IEEE Trans. Biomed. Eng..

[10] McDonald A.D., Schwarz C., Lee J.D., Brown T.L. (2012). Real-time detection of drowsiness related lane departures using steering wheel angle. Proceedings of the Human Factors and Ergonomics Society Annual Meeting.

[11] Ma J., Murphey Y.L., Zhao H. Real time drowsiness detection based on lateral distance using wavelet transform and neural network. Proceedings of the 2015 IEEE Symposium Series on Computational Intelligence.

[12] Kiashari S.E.H., Nahvi A., Bakhoda H., Homayounfard A., Tashakori M. (2020). Evaluation of driver drowsiness using respiration analysis by thermal imaging on a driving simulator. Multimed. Tools Appl..

[13] Bamidele A.A., Kamardin K., Abd Aziz N.S.N., Sam S.M., Ahmed I.S., Azizan A., Bani N.A., Kaidi H.M. (2019). Non-intrusive driver drowsiness detection based on face and eye tracking. Int. J. Adv. Comput. Sci. Appl..

[14] Khunpisuth O., Chotchinasri T., Koschakosai V., Hnoohom N. Driver drowsiness detection using eye-closeness detection. Proceedings of the 2016 12th International Conference on Signal-Image Technology & Internet-Based Systems (SITIS).

[15] Triyanti V., Iridiastadi H. (2017). Challenges in detecting drowsiness based on driver’s behavior. IOP Conf. Ser. Mater. Sci. Eng..

[16] Knapik M., Cyganek B. (2019). Driver’s fatigue recognition based on yawn detection in thermal images. Neurocomputing.

[17] Tayab Khan M., Anwar H., Ullah F., Ur Rehman A., Ullah R., Iqbal A., Lee B.H., Kwak K.S. (2019). Smart real-time video surveillance platform for drowsiness detection based on eyelid closure. Wirel. Commun. Mob. Comput..

[18] Lin S.T., Tan Y.Y., Chua P.Y., Tey L.K., Ang C.H. (2012). Perclos threshold for drowsiness detection during real driving. J. Vis..

[19] Rosebrock A. Eye Blink Detection with Opencv, Python, and Dlib. https://pyimagesearch.com/2017/04/24/eye-blink-detection-opencv-python-dlib/.

[20] Moujahid A., Dornaika F., Arganda-Carreras I., Reta J. (2021). Efficient and compact face descriptor for driver drowsiness detection. Expert Syst. Appl..

[21] Sri Mounika T., Phanindra P., Sai Charan N., Kranthi Kumar Reddy Y., Govindu S. (2022). Driver Drowsiness Detection Using Eye Aspect Ratio (EAR), Mouth Aspect Ratio (MAR), and Driver Distraction Using Head Pose Estimation. ICT Systems and Sustainability.

[22] Celecia A., Figueiredo K., Vellasco M., González R. (2020). A portable fuzzy driver drowsiness estimation system. Sensors.

[23] Popieul J.C., Simon P., Loslever P. Using driver’s head movements evolution as a drowsiness indicator. Proceedings of the IEEE IV2003 Intelligent Vehicles Symposium. Proceedings (Cat. No. 03TH8683).

[24] Coetzer R., Hancke G. Driver fatigue detection: A survey. Proceedings of the AFRICON 2009.

[25] Liu W., Qian J., Yao Z., Jiao X., Pan J. (2019). Convolutional two-stream network using multi-facial feature fusion for driver fatigue detection. Future Internet.

[26] Soukupova T., Cech J. Eye blink detection using facial landmarks. Proceedings of the 21st Computer Vision Winter Workshop.

[27] Maior C.B.S., das Chagas Moura M.J., Santana J.M.M., Lins I.D. (2020). Real-time classification for autonomous drowsiness detection using eye aspect ratio. Expert Syst. Appl..

[28] Al Redhaei A., Albadawi Y., Mohamed S., Alnoman A. Realtime Driver Drowsiness Detection Using Machine Learning. Proceedings of the 2022 Advances in Science and Engineering Technology International Conferences (ASET).

[29] Rasna P., Smithamol M. (2021). SVM-Based Drivers Drowsiness Detection Using Machine Learning and Image Processing Techniques. Progress in Advanced Computing and Intelligent Engineering.

[30] Saradadevi M., Bajaj P. (2008). Driver fatigue detection using mouth and yawning analysis. Int. J. Comput. Sci. Netw. Secur..

[31] Sahayadhas A., Sundaraj K., Murugappan M. (2012). Detecting driver drowsiness based on sensors: A review. Sensors.

[32] Ngxande M., Tapamo J.R., Burke M. Driver drowsiness detection using behavioral measures and machine learning techniques: A review of state-of-art techniques. Proceedings of the 2017 Pattern Recognition Association of South Africa and Robotics and Mechatronics (PRASA-RobMech).

[33] Dwivedi K., Biswaranjan K., Sethi A. Drowsy driver detection using representation learning. Proceedings of the 2014 IEEE International Advance Computing Conference (IACC).

[34] Dua M., Singla R., Raj S., Jangra A., Shakshi (2021). Deep CNN models-based ensemble approach to driver drowsiness detection. Neural Comput. Appl..

[35] Rosebrock A. Face Detection with Dlib (Hog and CNN). https://pyimagesearch.com/2021/04/19/face-detection-with-dlib-hog-and-cnn/.

[36] Kartynnik Y., Ablavatski A., Grishchenko I., Grundmann M. (2019). Real-time facial surface geometry from monocular video on mobile GPUs. arXiv.

[37] Weng C.H., Lai Y.H., Lai S.H. (2016). Driver drowsiness detection via a hierarchical temporal deep belief network. Proceedings of the Asian Conference on Computer Vision.

[38] Datahacker How to Detect Eye Blinking in Videos Using Dlib and Opencv in Python. https://datahacker.rs/011-how-to-detect-eye-blinking-in-videos-using-dlib-and-opencv-in-python/.

[39] Cech J., Soukupova T. (2016). Real-Time Eye Blink Detection Using Facial Landmarks.

[40] Bhesal A.D., Khan F.A., Kadam V.S. (2022). Motion based cursor for Phocomelia Users. Int. J. Emerg. Technol. Innov. Res..

[41] Taschenbuch Verlag Schiffman H. (2001). Sensation and Perception: An Integrated Approach.

[42] Maior C.B.S., Moura M.C., de Santana J., do Nascimento L.M., Macedo J.B., Lins I.D., Droguett E.L. Real-time SVM classification for drowsiness detection using eye aspect ratio. Proceedings of the Probabilistic Safety Assessment and Management PSAM 14.

[43] Keras Team, Keras Documentation: The Sequential Model. https://keras.io/guides/sequential_model/.

[44] Breiman L. (2001). Random forests. Mach. Learn..

[45] Scikit-Learn 1.4. Support Vector Machines. https://scikit-learn.org/stable/modules/svm.html.

[46] Brownlee J. How to Use ROC Curves and Precision-Recall Curves for Classification in Python. https://machinelearningmastery.com/roc-curves-and-precision-recall-curves-for-classification-in-python/.

[47] Kumar A., Patra R. Driver drowsiness monitoring system using visual behaviour and machine learning. Proceedings of the 2018 IEEE Symposium on Computer Applications & Industrial Electronics (ISCAIE).

[48] Chirra V.R.R., Uyyala S.R., Kolli V.K.K. (2019). Deep CNN: A Machine Learning Approach for Driver Drowsiness Detection Based on Eye State. Rev. D’Intell. Artif..

[49] Yu J., Park S., Lee S., Jeon M. (2019). Driver Drowsiness Detection Using Condition-Adaptive Representation Learning Framework. IEEE Trans. Intell. Transp. Syst..

[50] Fatima B., Shahid A.R., Ziauddin S., Safi A.A., Ramzan H. (2020). Driver fatigue detection using viola jones and principal component analysis. Appl. Artif. Intell..

[51] Ed-doughmi Y., Idrissi N., Hbali Y. (2020). Real-Time System for Driver Fatigue Detection Based on a Recurrent Neuronal Network. J. Imaging.

[52] Sheikh A.A., Mir J. Machine Learning Inspired Vision-based Drowsiness Detection using Eye and Body Motion Features. Proceedings of the 2021 13th International Conference on Information & Communication Technology and System (ICTS).

